# Supplementary figures and images for: Structural obstruction to full DNA replication in terminally differentiated skeletal muscle cells
Source: EMBO Rep. 2025 Aug 26;26(19):4633–55. doi: 10.1038/s44319-025-00554-x (PMC12508123; doi:10.1038/s44319-025-00554-x)

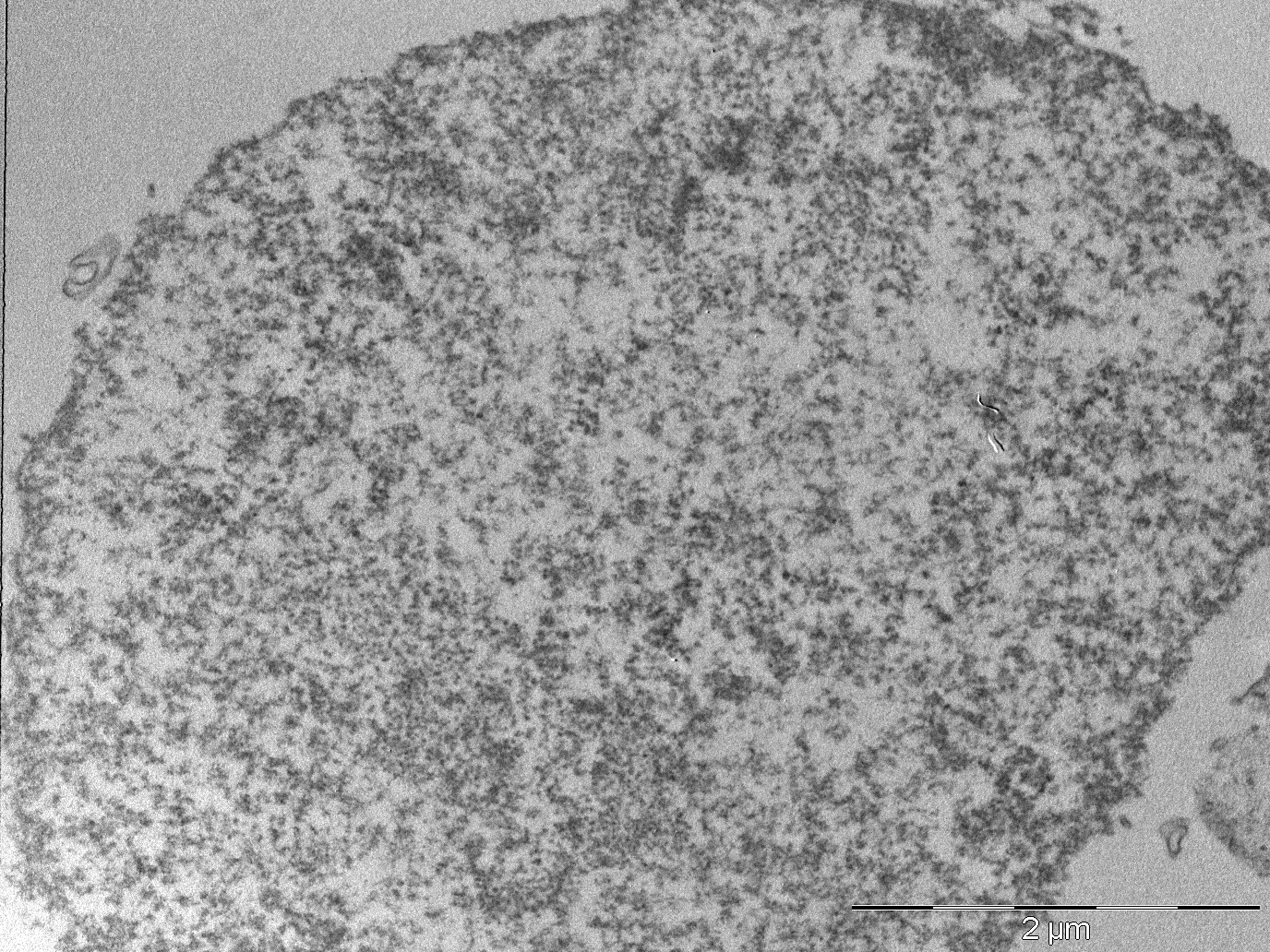

Supplement: Supplementary file 5 — Source data Fig. 3 [file 44319_2025_554_MOESM5_ESM.zip › Figure 3 source images/Fig 3C.tif]

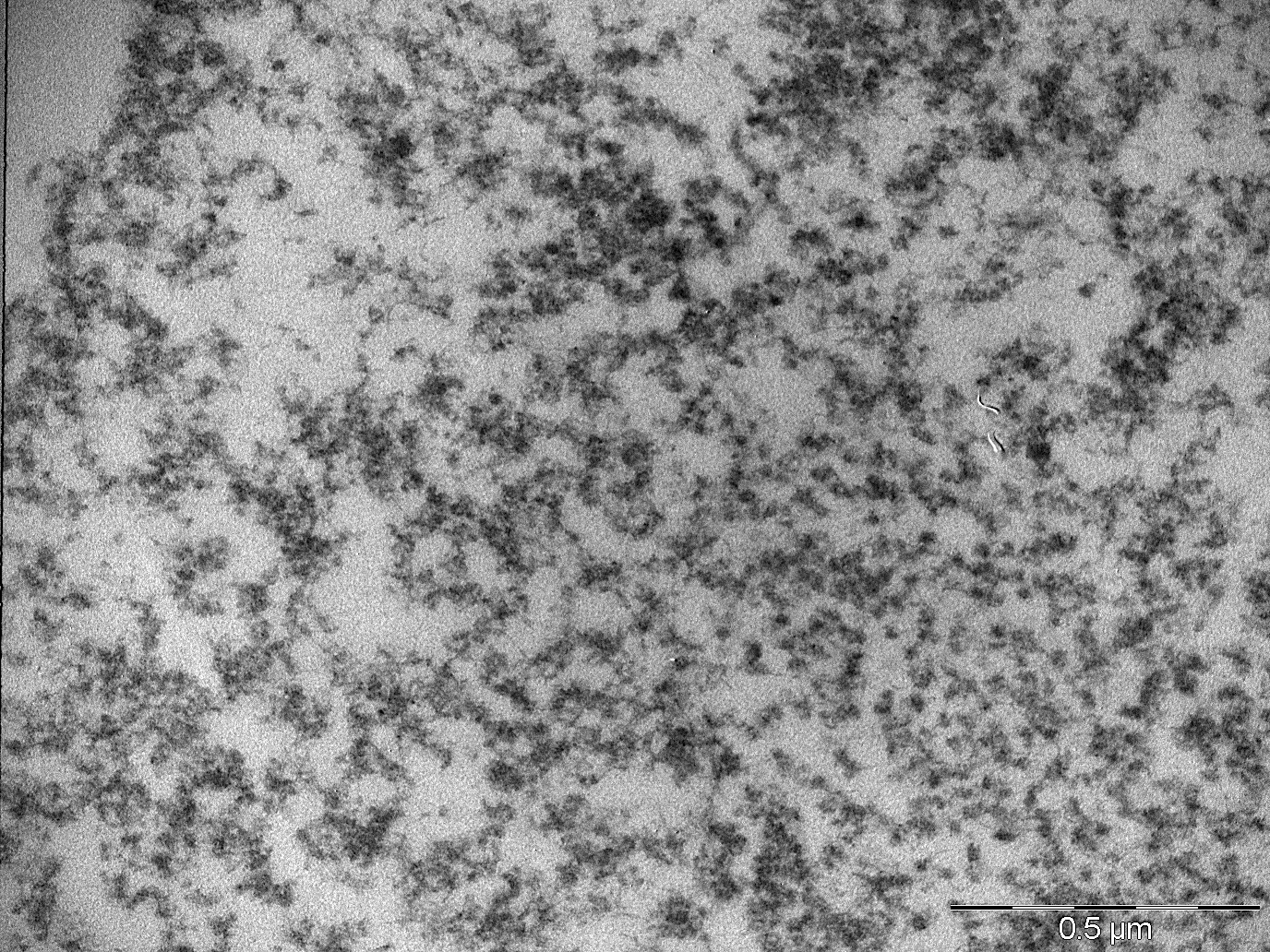

Supplement: Supplementary file 5 — Source data Fig. 3 [file 44319_2025_554_MOESM5_ESM.zip › Figure 3 source images/Fig 3D.tif]
