# Supplementary material for: Structural obstruction to full DNA replication in terminally differentiated skeletal muscle cells
Source: EMBO Rep. 2025 Aug 26;26(19):4633–55. doi: 10.1038/s44319-025-00554-x (PMC12508123; doi:10.1038/s44319-025-00554-x)
Supplement: Supplementary file 8 — Expanded View Figures [file 44319_2025_554_MOESM8_ESM.pdf]

Expanded View Figures

Figure EV1. Reactivation of DNA replication in MBs and MTs.

(**A**) MTs reactivated for the CGH experiments were continuously labeled with BrdU until harvest. Percentages of $BrdU^+$ MT are shown. (**B**) MBs made quiescent in suspension culture were either not replated (negative control) or replated in tissue culture dishes and pulse-labeled with BrdU for 30 min at the indicated times. Percentages of $BrdU^+$ MBs are shown. (**C**) Examples of immunofluorescence micrographs on which the data presented are based. Bars: 25 µm.

 © The Author(s)

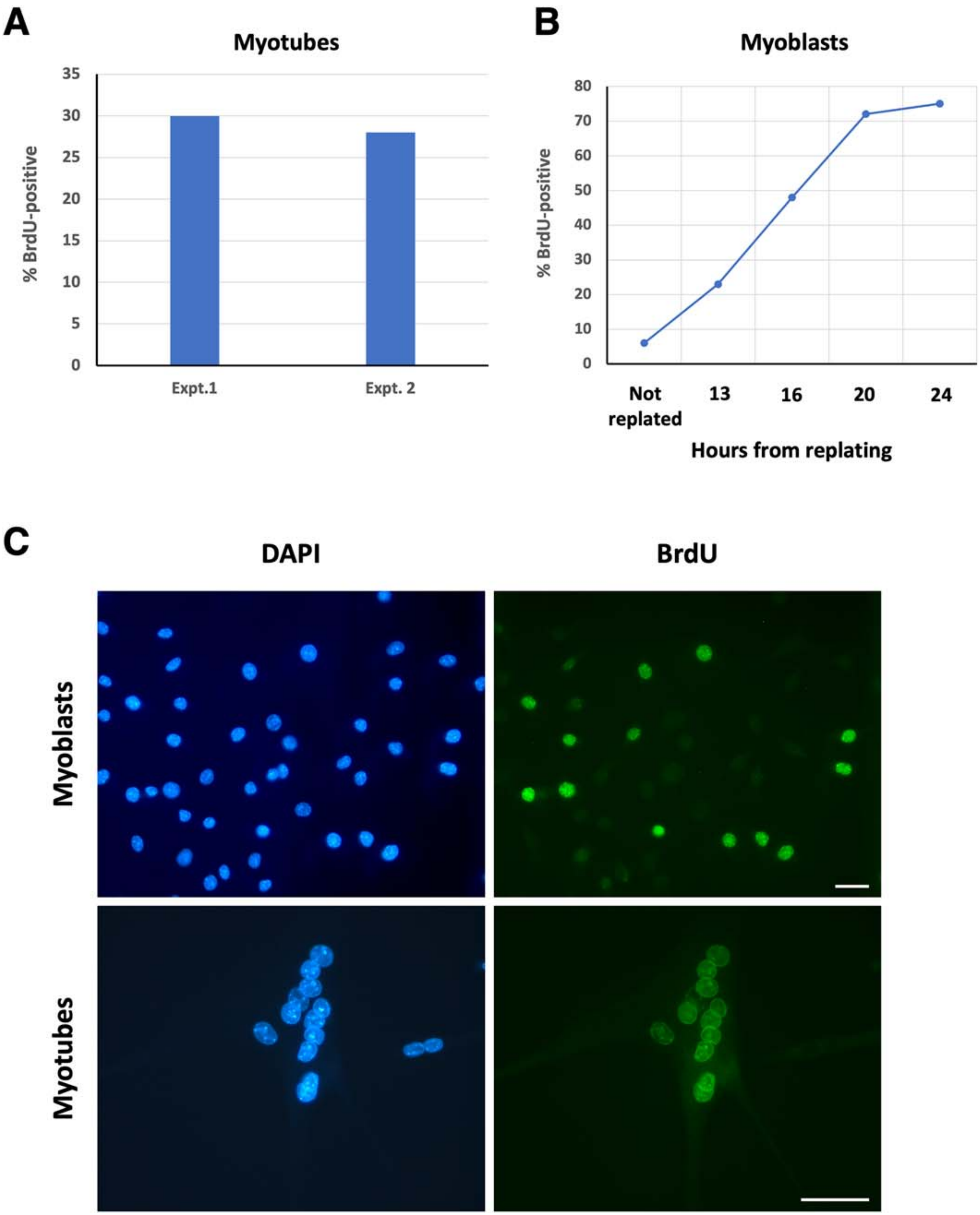
A
Myotubes
% BrdU-positive
35
30
25
20
15
10
5
0
Expt.1
Expt. 2
B
Myoblasts
% BrdU-positive
80
70
60
50
40
30
20
10
0
Not replated
13
16
20
24
Hours from replating
C
DAPI
BrdU
Myoblasts
Myotubes

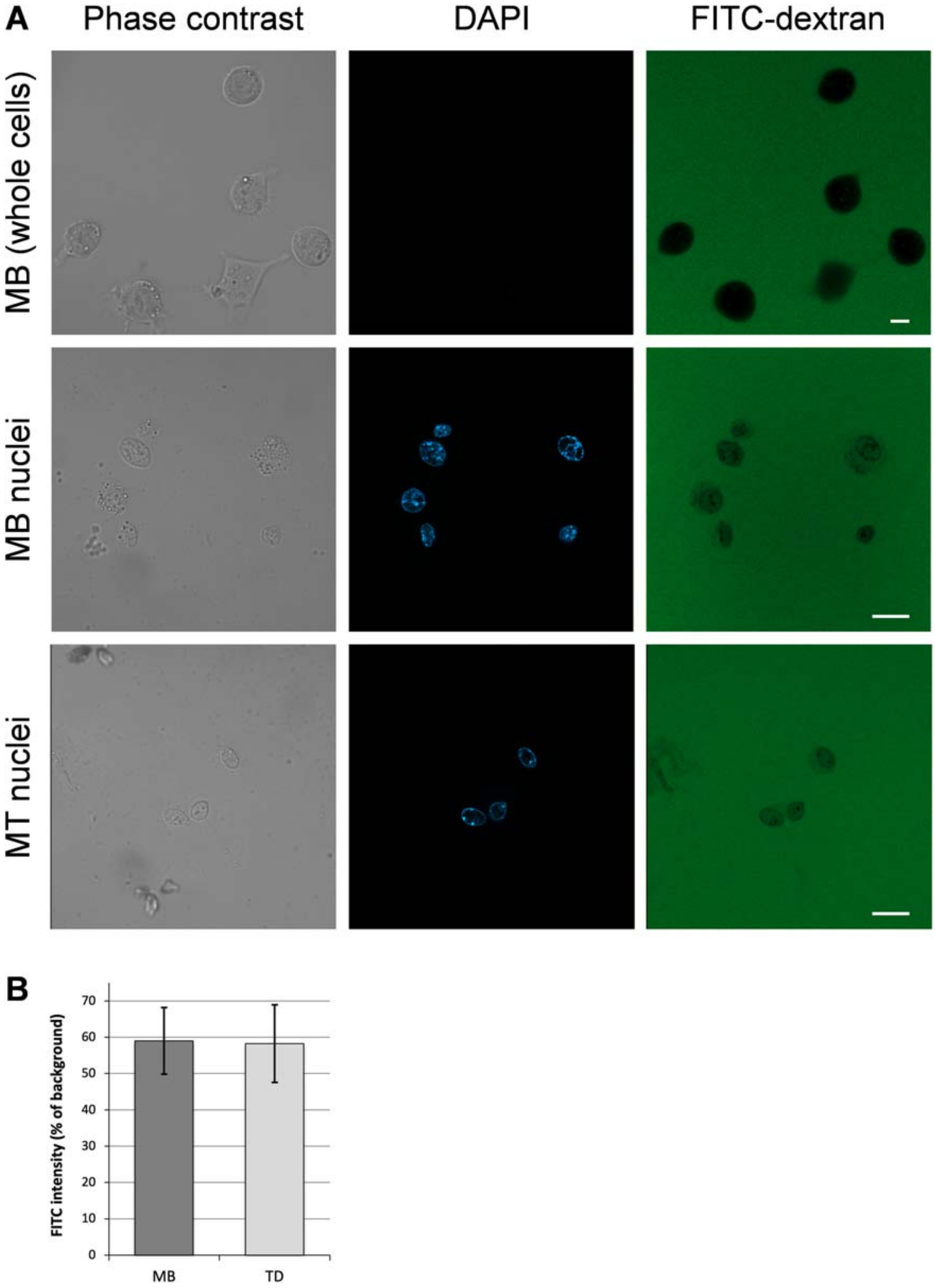

Figure EV2. Permeabilization assays.

(**A**) Whole, non-permeabilized MBs (negative control), MB nuclei, or MT nuclei were incubated in a DAPI/FITC-dextran mix, spread on glass slides and analyzed by confocal microscopy. A set of representative images is shown for each sample. (**B**) Dextran uptake in MB and MT nuclei, expressed as percentage of background fluorescence intensity, with standard deviation. Statistical analysis indicates no significant difference between the two samples MBs=59.0 ($n = 21$); MTs=58.2 ($n = 19$); unpaired t test, $P = 0.814$).

 © The Author(s)

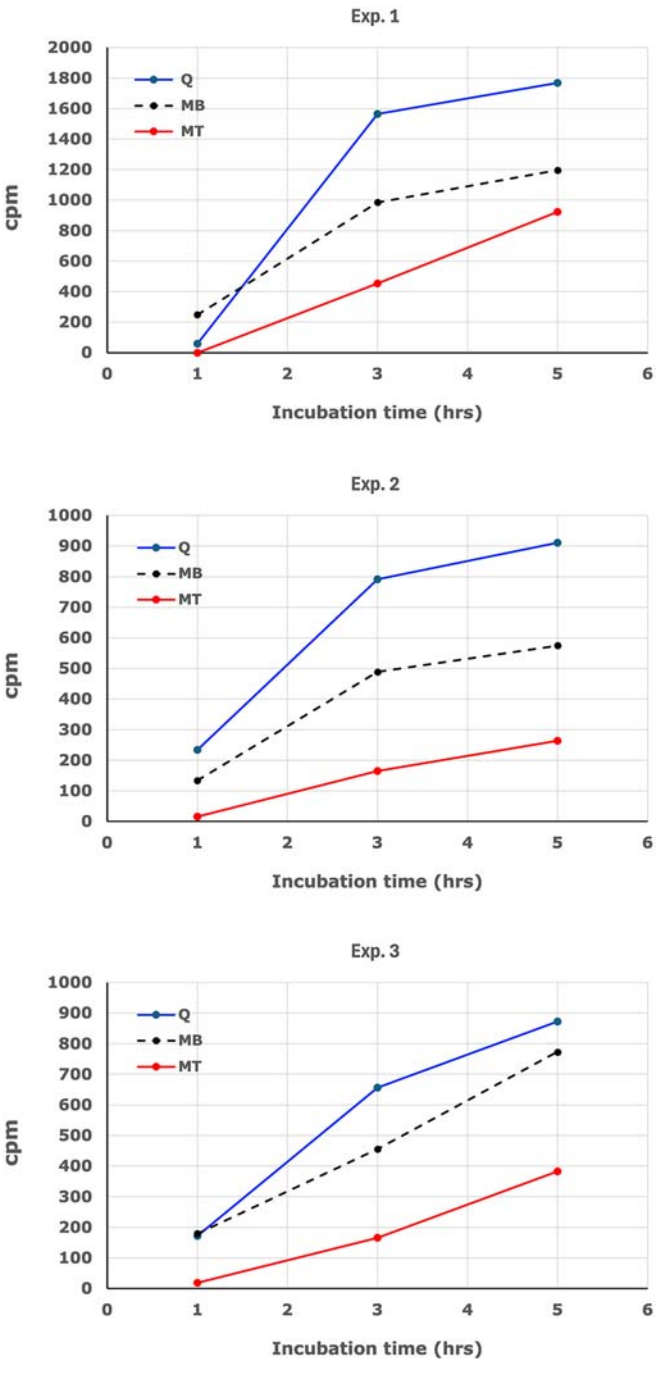

Figure EV3. ^{3}H TTP in MB and MT nuclei incubated in XEE.

Incorporation of ^{3}H-dTTP into nuclei incubated in XEE. The three experiments averaged in Fig. 2 are shown here separately. Disintegration counts per minute (cpm) were measured at the indicated incubation times and normalized to the number of nuclei in each reaction.

© The Author(s)

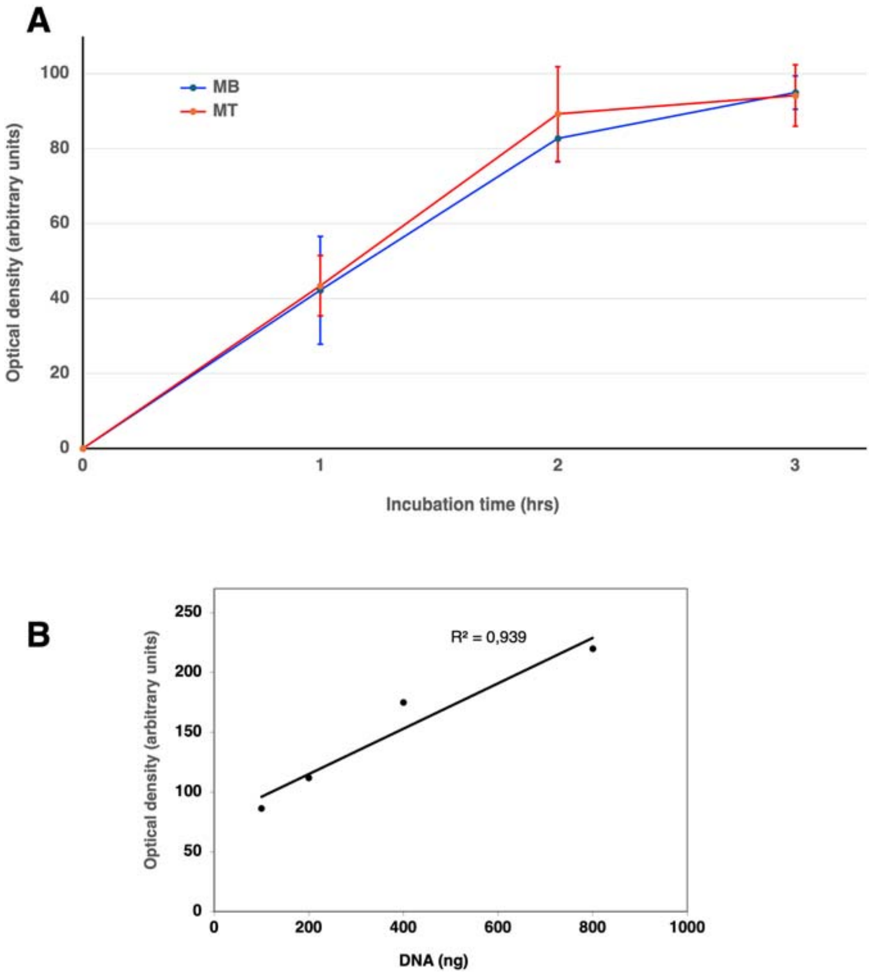

Figure EV4. Replication of purified DNA in XEE.

(**A**) Incorporation of biotin-16-dUTP into DNA purified from MBs or MTs in XEE. The average results of three independent experiments are shown, with standard deviations. Label incorporation was detected by dot blot, using horseradish peroxidase-conjugated streptavidin (see Methods and Protocols for details). The amount of DNA synthesized at the indicated times is expressed as the average optical density of three replicate spots from the same aliquot on the dot blot. (**B**) To ensure the accuracy and quality of the dot blots, each included a linear regression based on varying amounts of PCR-synthesized biotinylated DNA. An example from the second experiment is shown here. In the three experiments, the coefficient of determination (R^2) ranged from 0.9390 to 0.9737.

 © The Author(s)

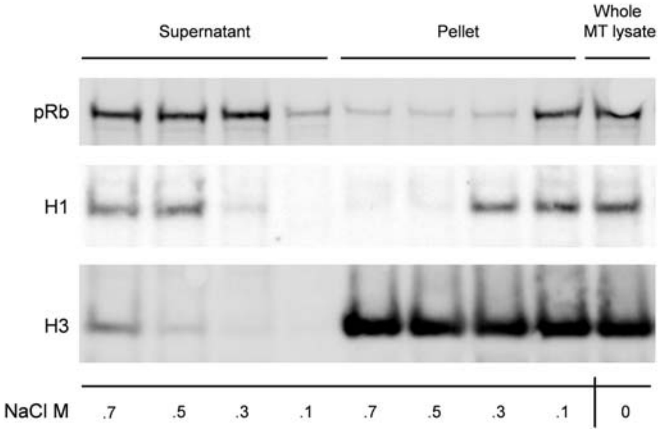

Figure EV5. NaCl concentration finder.

Nuclei isolated from MT were treated for 30 min with the indicated molar concentrations of NaCl and centrifuged at 500 g. The indicated proteins from supernatants and nuclear pellets were analyzed by western blotting. pRb: Retinoblastoma protein; H1: histone H1 (all subtypes); H3: histone H3 (all subtypes).

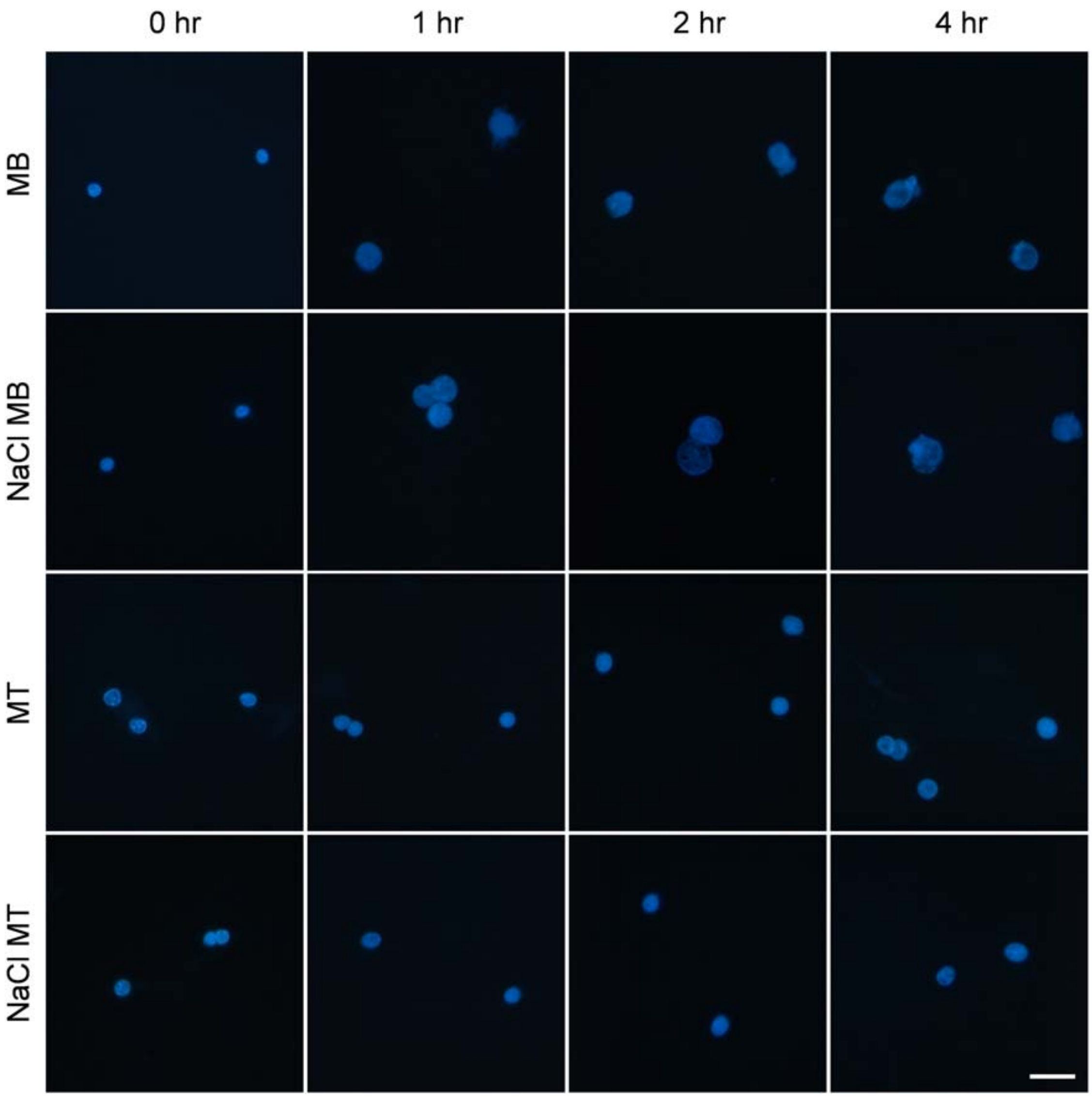

Figure EV6. Microscopy of MB and MT nuclei incubated in XEE.

Fluorescent microscopy photographs of MB and MT nuclei, treated or not with NaCl, incubated in XEE for the indicated times, and stained with DAPI. These are examples of the nuclei used for the microfluorimetric measurements shown in Fig. 5 and Fig. EV3. Bar: 25 µm.

 © The Author(s)

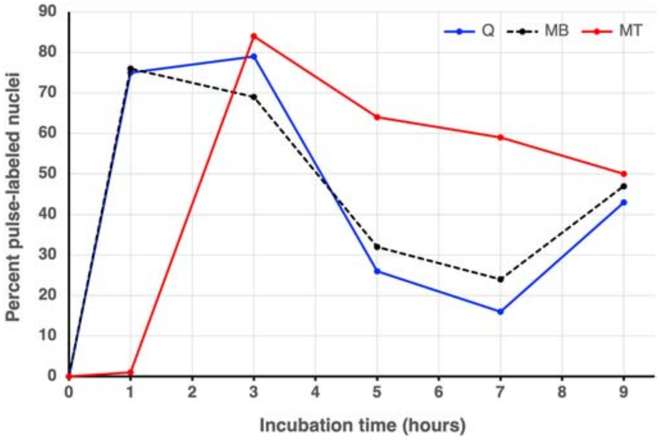

Figure EV7. Time course of pulse-labeled nuclei in XEE.

Nuclei from quiescent (Q) or proliferating MBs (MB) and non-reactivated myotubes (MT) were incubated in XEE in parallel. Thirty minutes before times 1, 3, 5, 7, and 9 h, one incubation mixture per nucleus type was pulse-labeled with Cy3-dCTP for 30 min before times 1, 3, 5, 7, and 9. The graph shows the percentages of Cy3-positive nuclei at each time point. Continuous Cy3 labeling throughout the experiment of an identical reaction set (three reactions: Q, MB, and MT) showed that all nucleus types were reactivated similarly: at the 5 h time point, Cy3-positive nuclei were: Q, 96%; MB, 95%; MT, 93%.
